# Effects of Community-Based Health and Social Interventions on Mental Health Outcomes Among People Experiencing Homelessness: A Systematic Review

**DOI:** 10.3390/nursrep16060202

**Published:** 2026-06-12

**Authors:** Elena Andina-Díaz, Bárbara Santamarta-Fernández, Elena Fernández-Martínez

**Affiliations:** 1 HeQoL Research Group, Department of Nursing and Physiotherapy, University of León, 24007 León, Spain; elena.andina@unileon.es (E.A.-D.); elena.fernandez@unileon.es (E.F.-M.); 2 CECAVIS Research Group, León Institute of Biosanitary Research IBIOLEÓN, Altos de Nava s/n, 24071 León, Spain; 3 EYCC Research Group, Department of Nursing, University of Alicante, San Vicente de Raspeig, 03690 Alicante, Spain; 4 University Hospital of León, Altos de Nava s/n, 24071 León, Spain

**Keywords:** homelessness, community-based interventions, mental health, psychosocial wellbeing, social determinants of health

## Abstract

**Background:** Community-based mental health and social interventions focusing on housing stability, integrated care and psychosocial support are being increasingly recognised as essential for improving the mental health and wellbeing of people experiencing homelessness. However, evidence regarding the effectiveness of these interventions remains fragmented across different models of care and study designs. This review synthesises how these interventions address mental health and social determinants of health. **Methods**: Following PRISMA 2020 guidelines, a systematic search of six electronic databases (2019–2025) was conducted (PROSPERO: CRD420250653260). The review included 29 quantitative, qualitative, and mixed-methods studies examining community-based interventions for people experiencing homelessness and mental health conditions according to predefined eligibility criteria. Methodological quality was assessed using the Mixed Methods Appraisal Tool. **Results**: Community-based interventions, particularly Housing First models, were frequently associated with improved housing stability, mental health outcomes, and programme retention. Integrated multidisciplinary services and outreach promote psychosocial wellbeing, continuity of care and reducing emergency service use. Peer-led programmes support social integration, although evidence regarding technology-based interventions was inconsistent, with some studies reporting improved engagement and access to support, while others found limited effects on mental health outcomes. **Conclusions:** Addressing social determinants of health through structured community-based interventions is essential to tackle mental health inequalities. The findings support the implementation of integrated community-based services combining housing, mental health, and social support. These results may inform policymakers, healthcare providers, and community organisations seeking to reduce mental health inequalities among people experiencing homelessness.

## 1. Introduction

Homelessness is a growing global public health concern, with the United Nations estimating that 150 million people are experiencing homelessness worldwide. In Europe, the situation is equally alarming. According to the 2024 European Federation of National Organisations Working with the Homeless (FEANTSA) report [1], approximately 1,287,000 people across Europe are homeless, including those sleeping rough, staying in night shelters, or living in temporary accommodations. Mental illness affects approximately 30–50% of people experiencing homelessness, often in combination with substance use and chronic physical health conditions [2]. Comorbid conditions, including substance use disorders, further complicate access to and effectiveness of mental healthcare and social interventions [2,3].

Homelessness encompasses diverse experiences, including chronic, episodic, and transitional forms, each associated with varying levels of housing instability and health vulnerabilities [4]. The relationship between homelessness and mental illness is bidirectional. According to a recent review [5], mental health conditions can contribute to housing instability, while homelessness exacerbates psychological distress, creating barriers to treatment and recovery. Social determinants of health, including poverty, unemployment, and lack of access to healthcare, further perpetuate this cycle [6]. As a result, interventions addressing both housing and mental health support are essential for sustainable improvements in wellbeing. Given the complex and multidimensional needs of people experiencing homelessness, mental health nurses play a key role in delivering community-based, trauma-informed, and recovery-oriented care through outreach, psychosocial support, therapeutic relationships, and care coordination [7,8,9].

Over the past two decades, recent reviews [10,11] have documented various interventions implemented to improve mental health outcomes for homeless individuals. Housing First (HF) programmes, which prioritise stable housing without preconditions, have gained extensive recognition for their effectiveness in reducing homelessness and improving their mental health [12]. Integrated healthcare services, including street medicine and mobile outreach programmes, aim to bridge the gap between healthcare providers and homeless individuals. Peer-support models and psychosocial interventions leverage community engagement to enhance service uptake and adherence [13]. Nurse-led interventions have also emerged as crucial components of care, addressing both physical and mental health needs through outreach and holistic support services [14].

Despite these efforts, challenges remain in evaluating the effectiveness of interventions and identifying best practices for long-term success. Studies often vary in their methodologies, making cross-comparisons difficult. Additionally, gaps in research exist regarding the sustainability of interventions, the role of health literacy, and the impact of social policies on homelessness and mental health [6,15]. A recent review highlights that access to mental health services and secure housing is critical for promoting recovery and social inclusion, reinforcing the importance of integrated strategies to address the complex needs of this population [5].

This review aligns with several Sustainable Development Goals (SDGs) [16], particularly SDGs 1, 3 10 and 11 by addressing mental health inequalities and access to care among vulnerable populations. Although previous systematic reviews have examined specific intervention models, particularly Housing First programmes and selected psychosocial approaches, evidence remains fragmented across the broader range of community-based interventions available for people experiencing homelessness and mental health conditions [2,5,12]. Existing reviews have rarely synthesised the effects of diverse intervention models within a single framework, limiting our understanding of how different approaches influence mental health outcomes, psychosocial wellbeing, housing stability, healthcare utilisation, and social integration among people experiencing homelessness with mental health conditions [5,12]. Consequently, a comprehensive synthesis of contemporary evidence is needed to improve our understanding of effective intervention components, inform service development, and support evidence-based community care.

Therefore, this systematic review aimed to synthesise existing the current evidence on the effectiveness of community-based mental health, social, and health interventions for people experiencing homelessness and mental health conditions. Specifically, the review examined the impact of these interventions on mental health outcomes, psychosocial wellbeing, housing stability, service utilisation, and social integration across diverse community and healthcare settings.

## 2. Materials and Methods

This systematic review was conducted following the Preferred Reporting Items for Systematic Reviews and Meta-Analyses (PRISMA) guidelines to ensure transparency and replicability [17]. The completed PRISMA 2020 checklist is presented in Supplementary Table S3. This review included studies from 2019 to 2025 that evaluated health and social interventions targeting homeless individuals with mental illness. The search was limited to studies published between 2019 and 2025 to capture contemporary evidence reflecting current community-based service models, including recent developments in Housing First programmes, integrated care approaches, and multidisciplinary mental health interventions. Restricting the review to the most recent literature was intended to enhance the relevance of findings for current policy and practice; however, it may have excluded influential earlier studies, particularly within the Housing First literature.

### 2.1. Literature Search

A comprehensive search was conducted in six major electronic databases: Web of Science, Scopus, PubMed, CINAHL, MEDLINE and PSICODOC. To improve reproducibility, the complete search strategy used in Web of Science is as follows: “homeless” OR “homeless*” OR “unhoused” OR “houseless” OR “rough sleep*” (Topic) and “community intervention*” OR “intervention*” OR “program*” OR “integrated care” OR “community treatment” OR “housing” OR “street outreach” OR “health intervention*” (Topic) and “mental health” OR “mental” OR “mental illness” OR “mental disorder*” OR “serious mental illness” and 2025 or 2024 or 2023 or 2022 or 2021 or 2020 or 2019 (Publication Years) and Review Article (Exclude—Document Types).

A broad definition of people experiencing homelessness was adopted. Definitions of homelessness were accepted as reported in the primary studies, including individuals living in shelters, temporary accommodations, unstable housing situations, or sleeping rough. The search terms included a combination of controlled vocabulary (MeSH terms) and free-text keywords related to homelessness, mental health disorders, community-based health and social interventions, and effectiveness outcomes. Boolean operators (AND, OR) were used to refine the search. Search strings were adapted to the indexing systems and syntax requirements of each database. Grey literature, conference proceedings, and unpublished studies were excluded to maintain methodological rigour.

The database searches were complemented by a manual screening of references from relevant systematic reviews and meta-analyses to ensure that no key studies were overlooked. Searches were limited to studies published in English between 2019 and 2025 to ensure relevance to contemporary intervention strategies.

The following criteria was followed in Table 1:

### 2.2. Data Retrieval

Studies meeting the predefined inclusion criteria were retrieved and assessed for eligibility. These criteria included intervention studies targeting people experiencing homelessness with mental health outcomes published between 2019 and 2025.

### 2.3. Screening and Selection

**Figure 1:**
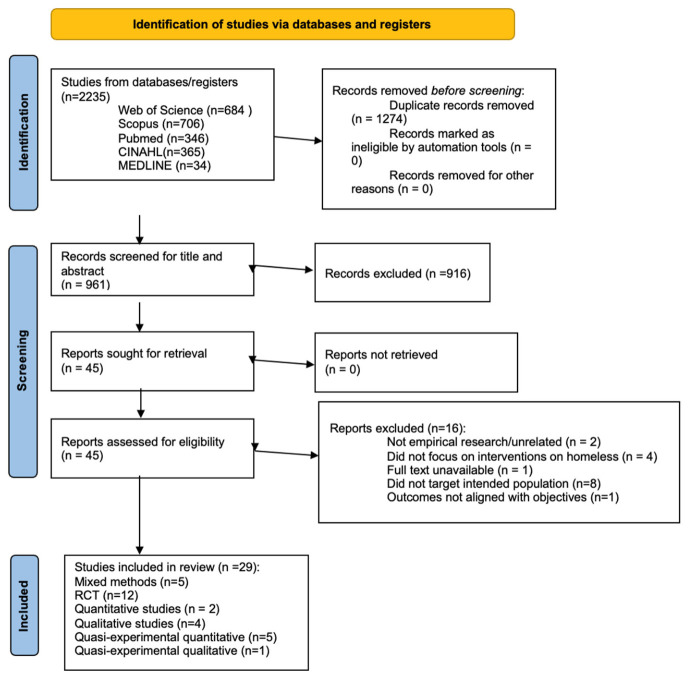
Prisma Flowchart [17].

Initially, 2235 references were screened, with 1274 duplicates removed, leaving 961 studies for title and abstract screening. Of these, 916 were excluded based on relevance to the research question. The remaining 45 studies were assessed for full-text eligibility by two reviewers (EF and EA). After a thorough evaluation, 16 studies were excluded: two were not empirical research, four did not focus on targeted interventions, one lacked full-text availability, eight did not target the intended population and one had misaligned outcomes. Ultimately, twenty-nine studies (5 mixed-methods (both quantitative and qualitative interventions), 12 RCTs, 2 quantitative, 4 qualitative, 5 quasi-experimental quantitative and 1 quasi-experimental qualitative) met all the inclusive criteria. The complete screening and selection process is illustrated in the PRISMA flowchart (Figure 1).

### 2.4. Data Extraction

Relevant information was directly input into a spreadsheet during this process. The extracted data consisted of publication details, study design, population characteristics, intervention type, primary outcomes measured and pertinent findings. A summary of the extracted data for each included study is presented in Supplementary Material Table S1. Additionally, Mixed Methods Appraisal Tool (MMAT) scores were recorded for each study [18]. Data were extracted independently by two reviewers; any discrepancies during the extraction process were resolved through discussion or by consulting a third reviewer.

### 2.5. Study Risk of Bias Assessment

To minimise potential bias, the study implemented various strategies: (a) the entire research team participated in refining the study protocol (CRD420250653260); (b) two researchers independently conducted the literature search; (c) data extraction was performed independently by two researchers, supervised by the senior researcher; (d) the methodological quality of the studies was assessed independently by two researchers; and (e) decisions at each stage were made collectively, with meetings held to decide on progression to the next stage.

### 2.6. Quality Appraisal of the Studies

The methodological quality of the included studies was assessed using the Mixed Methods Appraisal Tool (MMAT) [18]. All studies that met the eligibility criteria underwent methodological quality appraisal. Quality assessment was conducted to evaluate the methodological strengths and limitations of the included studies and to inform interpretation of the findings. The detailed results of the quality appraisal and the MMAT scores are displayed in Supplementary Material Table S2. Two independent researchers assessed the methodological quality of the studies, resolving any disagreements through discussion until consensus was achieved [19]. Overall, the methodological quality was variable. Eleven studies [20,21,22,23,24,25,26,27,28,29,30] achieved a perfect MMAT score of 100%, indicating high methodological rigour and minimal risk of bias, regardless of whether they employed qualitative, quantitative, or mixed-methods designs.

An additional nine studies [31,32,33,34,35,36,37,38,39] scored 80%, suggesting moderate to high quality with some minor limitations, such as unclear reporting of sampling methods or partial fulfilment of representativeness criteria. Nine studies [40,41,42,43,44,45,46,47,48]–scored between 40 and 60%, often due to incomplete reporting of participant follow-up, lack of justification for methodological choices, or weaknesses in outcome measures. These limitations were noted but did not significantly compromise their validity or reliability. No studies were excluded based on MMAT scores as studies with lower MMAT scores were retained to preserve the breadth of available evidence but their findings were interpreted with caution and considered within the context of their methodological limitations.

### 2.7. Data Analysis and Synthesis

A convergent integrated approach was employed to merge qualitative and quantitative data, including findings from mixed-methods studies [19]. In this systematic literature review, Mayring’s analysis (QCA, [49]) was utilised as a method to categorise and interpret textual data, identifying common patterns, themes, and implicit meanings. This approach facilitated the integration of heterogeneous evidence into common thematic categories while preserving the contextual richness of qualitative findings and enabling comparison across diverse study designs.

The QCA process involved numerous systematic steps: initially coding the text to identify themes and assign relevant excerpts was carried out, defining these themes to select pertinent material, revising categories and themes in relation to the research questions, conducting final coding to refine and develop main themes, and presenting the results in a narrative and summary form, offering a comprehensive overview of the findings [49]. Regarding the quantitative data, we applied qualitisation to translate the findings into narrative interpretations [19]. This was meant to minimise potential errors from assigning numerical values to qualitative data, ensuring a more accurate representation of the findings [50]. Themes were developed through iterative coding and categorisation of findings across the included studies. Similar intervention characteristics and outcomes were grouped into broader categories, which were reviewed and refined by the research team until consensus was reached. Quantitative findings were qualitised by transforming numerical outcomes into narrative descriptions of intervention effectiveness and subsequently integrating them with qualitative evidence within the thematic synthesis.

## 3. Results

A total of 2235 records were identified through database searching. Following screening and eligibility assessment, 29 studies met the inclusion criteria and were included in the review. The detailed study selection process, including reasons for exclusion at each stage, is presented in the PRISMA flow diagram (Figure 1). The 29 included studies represented diverse community-based health and social interventions targeting people experiencing homelessness and mental health conditions. Study designs included randomised controlled trials, qualitative studies, mixed-methods research, and quasi-experimental evaluations. Participants varied widely in age, gender, ethnicity, and mental health diagnoses, reflecting the heterogeneity of the population studied. Detailed study characteristics are presented in Table 1 and Supplementary Material Table S1.

The results of this review are structured around key themes identified from the content, allowing for a comprehensive synthesis of the evidence. The themes—Housing First, Integrated Healthcare Services, Technology-Based Interventions, Peer Support and Psychological Interventions, and Outreach programmes—were determined through consensus among the researchers to ensure analytical rigour and thematic coherence.

### 3.1. Housing First Interventions

Housing First (HF) programmes emerged as the most extensively studied intervention type across 11 included studies [22,23,25,26,28,30,32,35,40,47,48]. Overall, HF interventions were generally associated with improved housing stability, reductions in emergency department utilisation and psychiatric hospitalisations, and improvements in quality of life and psychosocial wellbeing. Several randomised controlled trials [22,23,47] reported significant reductions in emergency service use and improved retention rates among participants receiving HF interventions. Studies conducted in Canada, Spain, France, and the United States also demonstrated improvements in community integration, reductions in substance use, and increased access to mental health support services [25,28,32,34]. Martín-Fernández et al. [25] reported a 96.06% retention rate in a Spanish HF programme, alongside improvements in mental health status and quality of life. Long-term findings from Lachaud et al. [40] and Mejia-Lancheros et al. [26] suggested that HF interventions may reduce acute healthcare utilisation over time, although improvements in mental health outcomes and outpatient engagement remained variable. Studies focusing on specific populations, including women and individuals with cognitive impairment, further supported the feasibility and adaptability of HF models within diverse community settings [30,35]. Overall, the findings suggest that HF interventions are effective in promoting housing stability and service engagement, although their direct impact on mental health symptoms alone appears more inconsistent.

### 3.2. Integrated Healthcare Services

This category includes studies where the interventions are combined collaborations between professionals, facilities or health support systems, which continued over time. Chan et al. [21] evaluated an outpatient ambulatory intensive care unit model that provided intensive primary and behavioural healthcare to patients with unstable accommodation and complex needs. The study reported reductions in emergency department use and improvements in depression scores as well as an increase in primary care visits. Similarly, Arbour et al. [31] assessed a model integrating co-housing, psychiatric care, and case management, reporting significant improvements in both treatment adherence and housing stability. Vallesi et al. [48] reinforces this approach by combining medical, housing, and social services into a single coordinated team. Malden et al. [24], while focusing primarily on physical activity and peer support, incorporated mental health screening and referral processes, underlining the benefit of integrated support networks. Lowrie et al. [41] demonstrated that a pharmacist- and nurse-led outreach model, delivered at the point of hospital discharge, effectively bridged acute medical care with housing and social support, reducing readmissions and improving continuity for people experiencing homelessness.

### 3.3. Technology-Based Interventions

Two studies evaluated technology-mediated interventions. Nyamathi et al. [43] found modest improvements in stress levels through heart rate variability-biofeedback (HRV-BF), with Cohen’s d = 0.37 and health promotion reporting reductions in PTSD symptoms and improved mental wellbeing. However, Schueller et al. [46] examined mobile technology support programmes for homeless youth but reported no significant mental health improvements despite increased engagement and user satisfaction.

### 3.4. Peer-Support and Psychosocial Interventions

Several studies highlighted the importance of peer-support models in fostering engagement, reducing stigma, and improving trust in care providers. Malden et al. [24] reported improvements in self-esteem and social interaction among youth participants in peer-led physical activity and support groups. However, evidence of direct symptom reduction was limited, and studies varied widely in methodological rigour. Similarly, peer mentoring [20] enhanced service engagement and self-confidence among young adults. Integrated models combining housing, health, and social support demonstrated stronger mental health impacts. Parkes et al. [27] found that when support came from peers’ trust grew and engagement followed. This study evaluated a peer navigator harm reduction programme, reporting reduced substance use, improved trust in services, and enhanced mental wellbeing. Similarly, Holding et al. [33] and Isaak et al. [34] found peer support key in fostering service engagement and improving emotional wellbeing. Nyamathi et al. [43] implemented trauma-informed cognitive behavioural therapy (CBT) for women in shelters, finding reductions in PTSD symptoms and improved empowerment scores. Multi-component interventions like those studied by Vallesi et al. [48] and Savaglio et al. [36] focused on combining accommodation with psychosocial rehabilitation, achieving reductions in psychiatric symptoms, and improved social functioning. In Spain, Martín-Fernández et al. [25] evaluated psychosocial interventions in temporary housing settings, showing reductions in anxiety and improved quality of life, particularly when peer-led support took place. Santa María et al. [45] used a mindfulness intervention to obtain youth perspectives on group therapy. Participants reported benefits including emotional regulation, safety, and identity.

### 3.5. Outreach Programmes

Interventions offering financial incentives (e.g., for attending appointments) showed mixed effectiveness. Reid et al. [29] found that incentives increased short-term engagement but were insufficient for long-term behavioural changes in users unless it was paired with psychosocial support. Street medicine models, such as those described by Perna et al. [28], showed promise in enhancing access to care, especially for individuals with complex comorbidities. Wetherill et al. [38] examined the impact of offering nutritional interventions in a soup kitchen setting on attendees’ self-rated health and engagement, demonstrating that non-clinical programmes can also act as vital entry points to care. Health outreach programmes [28,38] improved physical and mental health service engagement but showed limited quantitative evidence for mental health symptom reduction. Lowrie et al. [41] also describes the use of community outreach visits, aligning with elements seen in dedicated outreach interventions. Similarly, Winiarski et al. [39] showed that bringing clinical support directly into shelters helped people who might otherwise go unseen to access mental healthcare or addiction treatment. Similarly, Savaglio et al. [36] reached individuals in urban areas who were disconnected from services, using mobile outreach teams that offered support where people felt safest. Together, all these studies highlight the role of outreach in overcoming access barriers through flexible, community-embedded approaches.

## 4. Discussion

The results of this systematic review confirm that interventions combining stable housing with integrated health and psychosocial services yield the most substantial benefits for homeless individuals with mental illness, though the extent and sustainability of these improvements vary. These findings also reinforce the importance of recovery-oriented and relationship-based approaches that are frequently associated with community mental health nursing practice. When compared to other reviews [51], this study reaffirms the key role of stable housing but highlights that mental health benefits are maximised when interventions are holistic. In many of the included studies, sustained engagement appeared to depend not only on housing provision itself, but also on continuity of care, therapeutic relationships, and ongoing psychosocial support. The effectiveness of community-based interventions may be explained by their ability to address both clinical needs and broader social determinants of health simultaneously. Stable housing, continuity of care, therapeutic relationships, and social support appear to operate synergistically, reducing barriers to service engagement and promoting recovery-oriented outcomes. These findings support ecological models of mental health, which emphasise the interaction between individual, social, and structural factors in shaping wellbeing.

The “Housing First” model remains the most evidence-based approach for improving housing stability, as consistently shown in recent reviews [11]. This review corroborates those findings, as studies like Tinland et al. [47] and Latimer et al. [23] also report consistent improvements in housing retention and reductions in emergency visits. Moreover, the findings regarding mental health symptom reduction were mixed. While some studies [32,40] found reductions in depressive symptoms and anxiety, others observed no significant changes in mental health despite stable housing [35]. The present review highlights the limited and inconsistent effects of housing alone on mental health symptoms, as noted in studies like O’Campo et al. [35] and Lachaud et al. [40], where housing alone did not consistently lead to substantial reductions in psychiatric symptoms, emphasising the need for adjunct mental health services.

Despite its strong evidence base, implementation of Housing First programmes may face important challenges, including high resource requirements, limited availability of affordable housing, variability in fidelity to the model, and difficulties sustaining long-term multidisciplinary support. These factors may partly explain the variability observed in mental health outcomes across studies and highlight the importance of adapting interventions to local service contexts.

Nurses are particularly well positioned to contribute to integrated and relationship-centred models of care for people experiencing homelessness and mental illness. Beyond clinical management, nursing practice can support care coordination, health promotion, and the development of trusting therapeutic relationships, which are recognised facilitators of engagement with healthcare services [28].

Additionally, there is diversity in the different profiles of the participants included in each study of the review, which underscores the complexity of health and social needs in homeless populations and highlights the need for tailored, culturally competent interventions.

Despite the evident importance of multidisciplinary approaches, our review identified a significant gap in the literature regarding nursing-led interventions. Only one study explicitly described a nurse-led model of care, despite mental health nurses being central to community outreach, therapeutic engagement, trauma-informed practice, and continuity of care for vulnerable populations. This underrepresentation is particularly concerning given the recognised role of mental health nurses in delivering recovery-oriented and person-centred care within community settings. This gap is particularly concerning given the key role of health professionals including nurses in delivering integrated, person-centred care, as emphasised by the International Council of Nursing [7] and the World Health Organization [8]. Strengthening the evidence base for nurse-led interventions is essential to inform practice and ensure that nurses are empowered to address the complex needs of this vulnerable population. Furthermore, investing in nursing leadership and research in this field aligns with the SDGs, particularly SDG 3 and SDG 10, which emphasise health equity and the reduction of disparities.

The findings suggest that community-based psychosocial and health-focused interventions may contribute to improvements in mental health outcomes among people experiencing homelessness, although the evidence remains limited and heterogeneous. Programmes such as mindfulness [45], biofeedback [43], and physical activity [24] were found to be feasible and acceptable but provided only modest symptom reduction, a finding consistent with other reviews [51], indicating that while mental health programmes for homeless populations are generally well-received, their effectiveness is limited by social instability, short intervention duration, and challenges in sustaining engagement.

Peer-delivered services were highlighted in the studies of Parkes et al. [27], Isaak et al. [34], and Holding et al. [33], which were found to be particularly effective in promoting trust-building, therapeutic engagement, service accessibility, and emotional wellbeing. These findings support earlier conceptual analyses on the benefits of peer involvement in mental healthcare and align with recent reviews emphasising the therapeutic value of peer support [5]. Collectively, they highlight the importance of relational, trust-building, and recovery-oriented approaches to care that are frequently embedded within community mental health nursing practice. These models may offer a relational element that is absent in purely clinical interventions, addressing barriers like stigma and mistrust that were identified in prior qualitative studies. Additionally, these results echo the findings of Carver et al. [52], who stressed the role of lived experience in fostering therapeutic alliances and addressing stigma in mental health service delivery for homeless populations. Differences in effectiveness across peer-support interventions may be related to variations in programme intensity, duration, integration with formal healthcare services, and the characteristics of participants. Interventions combining peer support with housing, case management, or mental health services generally demonstrated stronger outcomes than stand-alone peer programmes, suggesting that relational support alone may be insufficient to address complex and long-standing mental health needs.

Despite positive findings, the review identifies ongoing gaps in sustaining mental health improvements beyond housing outcomes, echoing concerns raised in previous work [11]. Interestingly, most studies reported follow-up periods of less than two years. However, the 7-year follow-up study by Mejia-Lancheros [26] offers a more comprehensive long-term perspective, demonstrating that while housing stability was largely maintained, but improvements in mental health outcomes were variable and sometimes diminished over time without sustained psychosocial support. This contrasts with the findings of Lachaud et al. [40], who reported reductions in emergency and inpatient care, highlighting that although HF programmes may decrease the use of acute services, it does not necessarily lead to increased engagement with outpatient care, as shown by the reduced primary and specialist visits observed by Mejia-Lancheros [26]. These finding suggest that chronic homelessness and mental illness require continuous, long-term interventions, and that short-term solutions are insufficient to address the enduring community-based health and social care needs of this population.

Importantly, several studies addressed subgroups such as youths [39,45,46] and women [34,35], who face additional barriers. Interventions tailored to homeless youths [39,45,46] emphasised the need for age-appropriate, engaging, and accessible approaches, such as mobile technology and mindfulness, though mental health improvements were moderate. Studies focusing on women [34,35] and the review “Interventions to improve the mental health of persons experiencing homelessness” [51] highlight the importance of gender-specific and trauma-informed care. These findings reinforce prior conclusions and advocate for case management, integrated services, and prioritisation of psychosocial wellbeing in homeless women and other vulnerable groups [2,12,51].

In terms of service delivery, this review highlights an increased use of community-based and outreach models, such as street medicine [28] and mobile mental health services [46]. These interventions meet individuals where they are, consistent with recommendations from the National Health Care for the Homeless Council [53] and others who advocate for low-threshold, harm-reduction-oriented services. The findings also resonate with discussions on social determinants of health addressing mental health in homelessness requires systemic interventions that go beyond clinical care, tackling poverty, trauma, and social exclusion. Interventions addressing only clinical aspects without tackling these broader determinants may achieve limited results.

Technology-based and incentive-driven interventions present opportunities for innovation, particularly in urban settings. Yet, current evidence suggests that these approaches require careful integration into broader support systems to achieve meaningful outcomes and they may not be appropriate for individuals with severe psychiatric symptoms or cognitive impairments [54].

Comparing methodologies across studies, RCTs provided the most robust evidence for housing interventions but often lacked qualitative insight into participants’ experiences. Conversely, qualitative and mixed-methods studies by Bell et al., Malden et al. and Parkes et al. [20,24,27] enriched the understanding of how participants experienced these interventions, particularly regarding the relational aspects of care.

### 4.1. Study Limitations and Recommendations

Firstly, the restriction to English-language publications may have introduced language bias and potentially excluded relevant studies published in other languages. Although study selection, data extraction, and quality appraisal were conducted independently by two reviewers, formal inter-reviewer agreement statistics (e.g., Cohen’s kappa) were not calculated. Therefore, the consistency of reviewer decisions cannot be quantitatively assessed. Several included studies demonstrated moderate methodological limitations, with MMAT scores ranging between 40% and 60%. These limitations were primarily related to incomplete reporting, participant attrition, limited justification of methodological choices, and weaknesses in outcome measurement. Although these studies were retained to provide a comprehensive overview of available evidence, their findings should be interpreted with caution and may reduce the certainty of conclusions regarding some intervention categories. Furthermore, substantial heterogeneity in study designs, intervention components, outcome measures, and follow-up periods limited direct comparisons across studies and precluded quantitative synthesis. This heterogeneity may have contributed to variations in reported effectiveness and should be considered when interpreting the generalisability of the findings. Definitions of homelessness varied across the included studies, and no standardised classification framework was used as an inclusion criterion. As a result, participants may have represented different forms and degrees of housing exclusion, potentially affecting comparability across studies and the interpretation of findings. Several critical gaps persist. First, long-term follow-up data are sparse. Most studies report outcomes over 6- to 24-month periods, limiting insights into sustainability. Second, cultural, gender, and age-specific needs are underexplored. Women, youth, and individuals with dual diagnoses may benefit from tailored interventions not yet reflected in the evidence base found. Third, health literacy—although recognised as a determinant of access and engagement—is rarely assessed [15]. This aligns with broader concerns about the lack of formal psychotherapeutic nursing roles in countries like Spain, where the implementation of structured advanced clinical practice could help address such gaps by improving communication, supporting patient understanding, and encourage sustained engagement [9]. Publication bias may also have influenced the findings, as studies reporting positive intervention outcomes are generally more likely to be published than studies reporting null or negative results. Consequently, the overall effectiveness of some intervention models may be overestimated. Additional limitations should be acknowledged. The exclusion of grey literature, conference proceedings, and unpublished studies may have resulted in the omission of potentially relevant evidence and contributed to publication bias. Furthermore, variability in the definitions of homelessness used across the included studies may have affected comparability, as not all studies employed a standardised framework such as the European Typology of Homelessness and Housing Exclusion (ETHOS) [55]. Finally, due to substantial heterogeneity in intervention types, study designs, outcome measures, and follow-up periods, a meta-analysis was not feasible. Consequently, the findings are based on narrative synthesis and should be interpreted with consideration of these methodological constraints.

### 4.2. Implications for Mental Health Nursing Practice and Research

This review underscores the essential role of psychiatric and mental health nurses in implementing integrated, trauma-informed, recovery-oriented, and community-based models of care for people experiencing homelessness and mental illness.

Multidisciplinary teams, including mental health nurses and allied professionals play a crucial role in delivering psychological support, outreach engagement, therapeutic relationships, continuity of care, and interdisciplinary coordination within holistic care models. Training in trauma-informed care, harm-reduction approaches, cultural competence, and case management is essential to support effective engagement with vulnerable populations experiencing homelessness and mental illness. Policy and service development should strengthen interdisciplinary collaboration, continuity of support, and accessible community-based care pathways.

The limited presence of explicitly nurse-led interventions identified in this review highlights a critical gap in current research. Given the central role of multidisciplinary teams in community and recovery-oriented practice, in which mental health nurses frequently play a key role, further evidence is needed to evaluate and strengthen collaborative models of care, including nursing-led approaches, that are aligned with global mental health and health equity goals [7].

## 5. Conclusions

This systematic review highlights the importance of integrated, community-based interventions in addressing the complex mental health and psychosocial needs of people experiencing homelessness. Housing First models consistently improved housing stability and engagement with support services, while integrated psychosocial, outreach, and peer-support interventions demonstrated potential benefits for psychosocial wellbeing, social integration, and continuity of care.

The findings suggest that interventions addressing both structural and psychosocial determinants of health are more effective than isolated psychosocial approaches. Recovery-oriented, trauma-informed, and person-centred models appear to be particularly important for sustaining engagement and promoting wellbeing among vulnerable populations experiencing homelessness and mental illness.

Mental health nurses are well positioned to support integrated and community-based approaches through outreach engagement, therapeutic relationships, continuity of care, and multidisciplinary collaboration. Future research should prioritise long-term follow-up studies and further evaluate nursing-led and trauma-informed interventions targeting diverse subgroups within homeless populations.

Ultimately, reducing mental health inequalities among people experiencing homelessness requires coordinated policies and community-based systems of care that prioritise stable housing, accessible mental health support, and meaningful social inclusion.

## Figures and Tables

**Table 1:** Inclusion criteria.

Criteria	Description
Population	People experiencing homelessness with mental health conditions
Intervention	Community-based health or social interventions
Outcomes	Mental health, psychological wellbeing, housing stability, quality of life
Study design	Quantitative, qualitative or mixed methods
Language	English
Timeframe	2019–2025

Developed by the authors based on the review objectives and eligibility criteria.

## Data Availability

All data analysed in this review are included within the manuscript, Supplementary Materials and referenced articles.

## Supplementary Materials

The following supporting information can be downloaded at https://www.mdpi.com/article/10.3390/nursrep16060202/s1, Table S1: Characteristics of included studies and data extraction summary; Table S2: Methodological quality appraisal of the included studies using the Mixed Methods Appraisal Tool (MMAT) version 2018; Table S3: Prisma Checklist 2020.

## Public Involvement Statement

There was no public involvement in any aspect of this research.

## Guidelines and Standards Statement

This systematic review was conducted and reported in accordance with the Preferred Reporting Items for Systematic Reviews and Meta-Analyses (PRISMA 2020) guidelines [17].

## Abbreviations

The following abbreviations are used in this manuscript:

CBT	Cognitive Behavioural Theory
ED	Emergency Department
ETHOS	European Typology of Homelessness and Housing Exclusion
HF	Housing First
MMAT	Mixed Methods Appraisal Tool
PRISMA	Preferred Reporting Items for Systematic Reviews and Meta-Analyses
PCP	Primary Care Physician
PTSD	Post-Traumatic Stress Disorder
QCA	Qualitative Content Analysis
RCT	Randomised Controlled Trial
SDGs	Sustainable Development Goals
